# Case-based learning in der Thoraxchirurgie: Ein Vergleich zwischen online- und präsenzbasiertem Unterricht

**DOI:** 10.1007/s00104-022-01626-7

**Published:** 2022-04-01

**Authors:** Katharina Rathberger, Michael Ried, Hans-Stefan Hofmann

**Affiliations:** Abteilung für Thoraxchirurgie, Universitätsklinikum Regensburg, Franz-Josef-Strauß-Allee 11, 93053 Regensburg, Deutschland

**Keywords:** CBL, Fallbasiertes Lernen, E‑Learning, Pulmonaler Rundherd, Lehrforschung, CBL, E‑learning, Learner-centered learning, Medical teaching, Pulmonary nodule

## Abstract

**Hintergrund:**

In der modernen medizinischen Lehre wird der klassische Frontalunterricht zunehmend durch innovative Lehrmethoden, wie z. B. „case-based learning“ oder E‑Learning (Electronic-Learning), ergänzt.

**Ziel der Arbeit:**

Konzipierung und Evaluation eines neuen Kurskonzeptes in der Thoraxchirurgie, um Studierende zu motivieren und zugleich Möglichkeiten zu finden, das ärztliche Personal in seiner Lehrtätigkeit zu entlasten.

**Material und Methoden:**

Alle Studierenden des jeweils 3. klinischen Semesters der Universität Regensburg absolvierten im Sommersemester 2016 und Wintersemester 2016/17 im Rahmen des Blockpraktikums Chirurgie ein Seminar nach dem Prinzip des fallbasierten Lernens zum Thema „Pulmonaler Rundherd“. Dabei wurde bei einer Gruppe von Studierenden ein moderiertes Präsenzseminar, bei der anderen eine reine Onlineveranstaltung abgehalten. Der Wissensgewinn und die subjektive Bewertung des Kurses durch die Studierenden wurden über Fragebögen ermittelt.

**Ergebnisse:**

Insgesamt nahmen 190 Studierende an den Seminaren teil, davon 88 am Präsenz- und 102 am Onlinekurs. Obwohl beide Gruppen einen deutlichen Wissenszuwachs durch die Kursintervention verzeichneten, zeigten die Studierenden des Präsenzkurses eine deutlich höhere subjektive Zufriedenheit im Vergleich zu ihren KommilitonInnen im Onlinekurs.

**Diskussion:**

Das fallbasierte Lernen erwies sich als Erfolg versprechendes Konzept in der thoraxchirurgischen Lehre, wobei sich die Etablierung von Onlinelernverfahren deutlich schwieriger gestaltete als bei präsenzbasiertem Unterricht.

## Abstract (en)

**Background:**

In modern medical education, traditional face-to-face teaching is increasingly being supplemented by innovative teaching methods, such as case-based learning or e‑learning.

**Aim of the work:**

Design and evaluation of a new course concept in thoracic surgery to motivate students and at the same time find ways to relieve the medical staff in their teaching activities.

**Material and methods:**

All students of the third clinical semester at the University of Regensburg completed a case-based course on the topic of pulmonary nodules in the summer semester 2016 and winter semester 2016/2017 as part of the practical course in thoracic surgery. This involved a moderated face-to-face seminar for one group of students and a purely online event for the other group. The knowledge gain and the subjective learning experience of the students were evaluated via questionnaires.

**Results:**

A total of 190 students took part in the courses, 88 in the face-to-face course and 102 in the online course. Although both groups recorded a significant increase in knowledge as a result of the course intervention, the students in the face-to-face course showed significantly higher subjective satisfaction compared to their peers in the online course.

**Conclusion:**

Case-based learning was shown to be a promising concept in thoracic surgery education, although establishing online learning procedures proved to be more challenging than presence-based teaching.

Der stetige Wandel in der Medizin birgt auch zahlreiche neue Herausforderungen für die medizinische Didaktik. Reines Fachwissen zu vermitteln, reicht nicht mehr aus – Teamwork, Selbstständigkeit, digitale Kompetenz und kreatives Denken gehören zu den Fähigkeiten, die sich junge Mediziner heutzutage aneignen müssen.

## Hintergrund

Die Fülle an medizinischem Wissen wächst rasant und hat sich im Jahr 2020 schätzungsweise alle 73 Tage verdoppelt [1]. Wissen, das sich Studierende im Verlauf ihres Studiums aneignen, ist daher oft schon bei ihrem Studienabschluss nicht mehr aktuell. Die zunehmende Interdisziplinarität und der mündige, gut informierte Patient lassen darüber hinaus vermehrt auch zwischenmenschliche Kompetenzen in den Vordergrund rücken [2]. Diesen Umständen muss auch in der Ausbildung des medizinischen Nachwuchses Rechnung getragen werden. Es gilt, den Fokus auf die Vermittlung von Lerntechniken und Förderung prozessorientierten Denkens statt auf reine Wissensinhalte zu legen. Die SARS-CoV-2-Pandemie („severe acute respiratory syndrome coronavirus type 2“) hat noch einmal zusätzlich deutlich gemacht, wie wichtig digitales Lernen und Arbeiten ist, um die medizinische Lehre zukunftsfähig zu machen [3].

Gleichzeitig fehlt es in Deutschland an chirurgischem Nachwuchs bei zugleich steigendem Versorgungsbedarf [4]. In einer Studie des Berufsverbandes Deutscher Chirurgen e. V. gaben 80 % der befragten chirurgischen Chef- und Oberärzte an, einen numerischen Bewerbermangel wahrzunehmen und sogar 94 % sahen einen qualitativen Bewerbermangel in ihrem Fachgebiet [5]. Das Interesse an chirurgischen Fächern ist zu Beginn des Studiums zunächst hoch, nimmt jedoch kontinuierlich ab und am Ende des praktischen Jahres streben nur noch 5 % der AbsolventInnen diese Weiterbildungsrichtung an [6]. Daher ist es für die Zukunft der Chirurgie essenziell, Medizinstudierende durch motivierende und praxisbezogene didaktische Konzepte schon früh für dieses Fach zu begeistern und als ärztlichen Nachwuchs zu gewinnen [7].

## Material und Methoden

### Studienkonzept

Für diese prospektive Beobachtungsstudie liegt ein positives Votum der Ethikkommission Regensburg vor. Alle Studierenden, die im Sommersemester 2016 und Wintersemester 2016/17 das 3. klinische Semester ihres Medizinstudiums am Universitätsklinikum Regensburg absolvierten, nahmen im Rahmen des Blockpraktikums Herz-Thorax-Chirurgie an einem „Case-based-learning“(CBL)-Kurs zum Thema „Pulmonaler Rundherd“ teil. Der Aufbau des Seminars, die Schwerpunkte sowie die dazugehörigen Lehrinhalte wurden durch ärztliche Mitarbeiter der Abteilung für Thoraxchirurgie definiert. Darauf basierend wurde eine Präsentation erstellt, welche das Wissen anhand eines klinischen Falles vermitteln sollte. In einer Gruppe wurde ein Präsenzseminar mit studentischem Tutor, in der anderen ein Onlinekurs mit dem CASUS®-Lernsystem durchgeführt. Die Zuteilung zu den beiden Gruppen erfolgte nach dem Zufallsprinzip und konnte durch die Studierenden nicht beeinflusst werden. Vor und nach dem Kurs erfolgte jeweils eine objektive Evaluation des Lernerfolgs mithilfe eines Wissenstest, bestehend aus zwei frei zu beantwortenden Fragen und drei Multiple-Choice-Fragen. Außerdem wurden Fragen zum Vorwissen im Fachgebiet Thoraxchirurgie und zur Vorbereitung auf den Kurs gestellt. Um die Ergebnisse der einzelnen Fragen des Wissenstests zusammenzufassen und sie in einen Zusammenhang mit den Ergebnissen der Evaluation setzen zu können, wurden die Einzelergebnisse zusammengezählt und daraus ein Summenscore ermittelt. Zur Quantifizierung des Wissenszuwachses, den die Probanden durch den Kurs insgesamt hatten, wurde die Differenz aus dem Score des Wissenstests vor und nach dem Kurs für beide Kursformate berechnet.

Zusätzlich konnten die Studierenden das Seminar subjektiv evaluieren. Die Fragen orientierten sich dabei an der Kurzform des „VB-Psych“, einem ursprünglich für die medizinische Psychologie entwickelten Testverfahren, und umfassten die Bereiche „Lehrmedium und Lernumgebung“, „Kursinhalte“ und „Vergleich mit anderen Lehrformaten“ [8]. Die Fragen waren auf einer Likert-Skala von 1 (stimmt nicht) bis 5 (stimmt sehr) zu beantworten.

### Zielsetzung

Mit dem CBL erprobten wir ein lernzentriertes, motivierendes Lehrkonzept im Rahmen des thoraxchirurgischen Kurrikulums am Universitätsklinikum Regensburg. Durch den Vergleich zwischen online- und präsenzbasiertem CBL sollten Erkenntnisse für eine zukunftsorientierte medizinische Lehre gewonnen werden.

### Statistische Analyse

Für die Analyse wurde die Software IBM® SPSS® Statistics 24 (Chicago, IL, USA) verwendet. Die Ergebnisse von Wissenstest und Evaluation und ihre Verteilung in den beiden Kursformaten wurden mit Kreuztabellen ermittelt und mittels χ^2^-Test auf Unabhängigkeit überprüft. Als Signifikanzniveau wurde für alle Tests *p* < 0,05 festgelegt.

## Ergebnisse

Das Probandenkollektiv der Studie bestand aus insgesamt 190 Studierenden des 3. klinischen Semesters. Davon nahmen 88 StudentInnen am Präsenzkurs und 102 am Onlineseminar teil. Das mittlere Alter betrug bei beiden Lehrformaten 23,8 Jahre. Etwa 56 % der Studierenden des Präsenzkurses und 65 % der Onlinekursteilnehmer waren weiblich.

**Figure 1:**
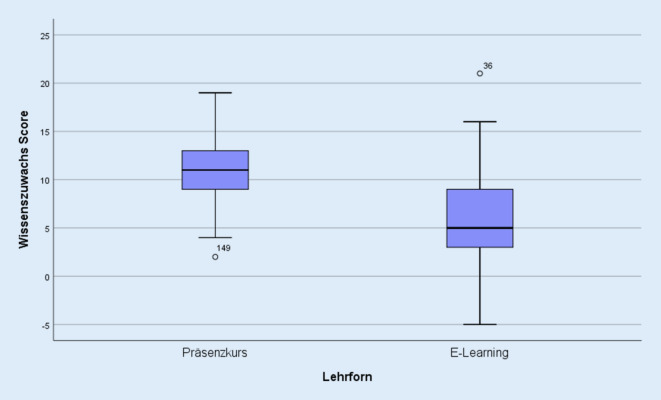

Die Probanden des Präsenzkurses erzielten beim Summenscore zur Ermittlung des Wissenszuwachses im Wissenstest im Mittel einen Differenzwert von 10,93 Punkten (±3,55). Die Onlinekursteilnehmer verbesserten sich um durchschnittlich 5,99 Punkte (±4,20; Abb. 1). Weder das Geschlecht der Studierenden noch die Vorbereitung auf den Kurs hatten einen signifikanten Einfluss auf das Ergebnis des Wissenstests.

Die Lernförderlichkeit des Tutors bewerteten die Studierenden des Präsenzseminars bei der ersten Frage der Evaluation mit durchschnittlich 4,74 (±0,51) Punkten auf einer Skala von 1 bis 5. Außerdem sollte die Zufriedenheit der Probanden mit ihrem jeweiligen Kursformat evaluiert werden. Etwa 95 % der Präsenzkursteilnehmer stimmten der Aussage zu, dass die Kleingruppe ein gutes Lehrformat für einen PBL-Kurs ist. Sie bewerteten diese Frage mit durchschnittlich 4,63 (±0,65) Punkten. Von den Studierenden des Onlinekurses waren ca. 78 % mit ihrem Kursformat zufrieden. Sie vergaben durchschnittlich 4,12 (±1,14) Punkte bei dieser Frage. Die Lernumgebung bewerteten die Studierenden im Präsenzkurs im Rahmen der dritten Frage im Mittel mit 4,72 (±0,70) Punkten. Bei ihren Kommilitonen im Onlinekurs waren es durchschnittlich 4,04 (±0,92).

In Tab. 1 sind die Fragen zum Thema „Kursinhalte“ und die jeweiligen deskriptiven Daten dargestellt. Insgesamt wurde der Präsenzkurs hier statistisch signifikant positiver bewertet als der Onlinekurs.

**Table 1:** 

Frage		Präsenzkurs	Onlinekurs	*p*-Wert
1. Ich hatte Spaß an der Bearbeitung des Falls	MW	4,27	3,35	< 0,001
	SD	±0,64	±1,00	
2. Ich konnte aktiv viel zur Bearbeitung des Falls beitragen	MW	3,80	3,10	< 0,001
	SD	±0,84	±0,98	
3. Der bearbeitete Fall war fachlich zu anspruchsvoll	MW	2,13	2,85	< 0,001
	SD	±1,15	±1,07	
4. Der heutige Fall war nah an der klinischen Realität	MW	4,42	4,07	0,002
	SD	±0,66	±0,82	
5. Die Struktur des Falls war für mich gut nachvollziehbar	MW	4,54	4,03	< 0,001
	SD	±0,67	±0,83	
6. Ich habe in diesem Kurs viel dazugelernt	MW	4,04	3,41	< 0,001
	SD	±0,72	±0,91	
7. Ich habe das behandelte Krankheitsbild verstanden	MW	4,44	3,61	< 0,001
	SD	±0,75	±0,95	
8. Ich konnte in diesem Kurs meine Fähigkeiten zur Bearbeitung klinischer Fälle erweitern	MW	3,87	3,41	< 0,001
	SD	±0,72	±0,92	
9. Ich fühle mich gut vorbereitet, wenn ich im klinischen Alltag auf einen ähnlichen Fall treffe	MW	3,16	2,52	< 0,001
	SD	±0,94	±0,98	

*MW* Mittelwert, *SD* Standardabweichung

Abschließend sollten die Teilnehmer das Lehrformat des CBL im Vergleich zu klassischem, frontal gehaltenem Unterricht bewerten. Hier vergaben die Teilnehmer für die Aussage, dass mehr Kurse in ihrem Studiengang nach dem Prinzip des CBL abgehalten werden sollten, durchschnittlich 4,40 ± 0,74 Punkte im Präsenzkurs bzw. 3,53 ± 1,22 Punkte im Onlinekurs. In beiden im Rahmen der Studie gelehrten Kursformaten bewerteten die Studierenden das CBL gut im Vergleich zu klassischen Unterrichtsformen, die Teilnehmer des Präsenzkurses zeigten sich jedoch bezüglich der Vorteile deutlich überzeugter als ihre Kommilitonen des Onlineseminars (Tab. 2).

**Table 2:** 

Frage		Präsenzkurs	Onlinekurs	*p*-Wert
14. Ich habe beim heutigen Kurs mehr gelernt als bei einem klassischen (frontal gehaltenen) Seminar	MW	4,13	3,02	< 0,001
	SD	±0,80	±1,25	
15. Ich denke, das Wissen aus dem heutigen Kurs werde ich mir besser merken können als bei klassischen (frontal gehaltenen) Seminaren	MW	4,15	3,05	< 0,001
	SD	±0,74	±1,23	
16. Ich fand den heutigen Kurs interessanter als klassische (frontal gehaltene) Seminare	MW	4,32	3,03	< 0,001
	SD	±0,72	±1,19	

*MW* Mittelwert, *SD* Standardabweichung

## Diskussion

Anhand eines Präsenz- und eines Onlinekurses sollte im Rahmen dieser Studie das Prinzip des „case-based learning“ innerhalb eines thoraxchirurgischen Kurrikulums erprobt werden. Die verschiedenen Lehrformate wurden sowohl subjektiv als auch objektiv evaluiert. Darüber hinaus konnten zahlreiche Erkenntnisse zur Integration fallbasierter Kurse in ein bestehendes chirurgisches Kurrikulum gewonnen werden.

### Objektive Evaluation

In den vor und nach den Kursen durchgeführten Wissensüberprüfungen zeigte sich in beiden Kursgruppen ein deutlicher Wissenszuwachs. Der Großteil der bisher durchgeführten Studien in der Lehrforschung überprüfte bisher nur das Wissen der Teilnehmer nach der Kursintervention. Die wenigen Forschungsarbeiten, die sowohl prä- als auch postinterventionelle Wissenstests durchführten, um den Wissenszuwachs abzubilden, zeigten jedoch ebenfalls einen durchweg positiven Effekt von CBL-Kursen auf das Wissen der Lernenden [9–11]. In der von uns durchgeführten Studie zeigte sich allerdings, dass die Studierenden des Präsenzkurses im Wissenstest fast ausschließlich signifikant bessere Resultate als ihre KommilitonInnen im Onlinekurs erzielten. Dies steht eher konträr zu den wenigen Studien in der medizinischen Lehrforschung, die den Wissensgewinn bei CBL-Kursen untersucht und festgestellt haben, dass Onlinelehrangebote sich vergleichbar oder teilweise sogar positiver als Präsenzkurse auf Wissen und Fähigkeiten von Medizinstudierenden auswirken [12, 13]. Die möglichen Ursachen für das unerwartet negativere Ergebnis der Studierenden des E‑Learning-Kurses sollen im Rahmen der Diskussion der subjektiven Evaluation noch genauer beleuchtet werden.

### Subjektive Evaluation

In allen drei Kategorien der Evaluation („Lehrmedium und Lernumgebung“, „Kursinhalte“ und „Vergleich mit traditionellen Lehrformen“) wurde das Präsenzformat im Vergleich zum Onlinekurs von den Studierenden fast durchgehend positiver bewertet. Besonders überraschte hier, dass gerade die Kursumgebung im Onlinekurs signifikant negativer bewertet wurde als im Präsenzkurs. Dies steht der Tatsache entgegen, dass eine zeitlich und örtlich individuell gestaltbare Lernsituation zu den klaren Vorteilen des E‑Learning gezählt wird [14]. Die Ursache für die unerwartet negative Einstellung der Teilnehmer gegenüber dem E‑Learning-Format könnte sein, dass dieses im Gegensatz zum Präsenzunterricht in Kleingruppen bisher kein fester Bestandteil des universitären Kurrikulums war. Diese Annahme würde auch durch weitere Evaluationsergebnisse der vorliegenden Studie unterstützt werden: Die meisten Teilnehmer des Präsenzkurses bewerteten die Frage, ob sie sich vorstellen könnten, einen Fall ähnlich gut am Computer bearbeiten zu können, im Durchschnitt neutral (2,87 Punkte [±1,10] auf einer Skala von 1 bis 5) und auch im Onlinekurs standen die Teilnehmer der Frage, ob mehr Kurse online abgehalten werden sollen, im Mittel neutral gegenüber (3,04 Punkte [±1,36] auf einer Skala von 1 bis 5). Dem entgegen stehen zahlreiche Studien, die keine negative Grundeinstellung von Mitarbeitern im Gesundheitswesen und insbesondere Medizinstudierenden gegenüber E‑Learning nachweisen konnten [15, 16]. Auch bezüglich der Kursinhalte evaluierten die Studierenden das präsenzbasierte Format deutlich positiver. Der fördernde Effekt, den CBL auf die Motivation und Wissen der Lernenden haben kann, ist gut belegt [10, 17]. Insbesondere bei E‑Learning-gestützten CBL-Formaten finden sich jedoch auch vereinzelte Studien, die gegenteilige Effekte aufzeigen konnten: Im von Nicklen et al. durchgeführten Vergleich von präsenz- und onlinebasiertem CBL evaluierten die Studierenden im E‑Learning-Kurs ihr Lehrformat deutlich schlechter und führten dies vor allem auf technische Probleme, den erhöhten Zeitaufwand und den Mangel an sozialer Interaktion in diesem Lehrformat zurück [13]. Thistlewaite et al. identifizierten in ihrem Review technische Fehlfunktionen und eine erschwerte Navigation auf der E‑Learning-Plattform als negativen Einflussfaktor auf das Evaluationsergebnis [10]. Es lässt sich also zusammenfassen, dass die Ergebnisse von Evaluation und Wissenstest im Onlinekurs im direkten Vergleich mit dem präsenzbasierten Kurs etwas schlechter waren. Eine Ursache könnte sein, dass digitale Lehrangebote zu diesem Zeitpunkt noch nicht regelmäßig im chirurgischen Kurrikulum integriert waren und daher noch keine ausreichende Akzeptanz bei den Studierenden fanden. Ebenso hat es seit Durchführung dieser Untersuchung eine deutliche Zunahme onlinebasierter Lehre sowie eine Verbesserung der technischen Voraussetzungen gegeben, welche v. a. auch durch fehlende Präsenzunterrichte im Rahmen der COVID-19-Pandemie erklärbar sind.

## Zusammenfassung und Ausblick

Unsere Untersuchungen konnten zeigen, dass fallbasiertes Lernen in der Thoraxchirurgie ein Erfolg versprechendes Konzept ist, das im Vergleich zu traditionellen Lehrformen von den Lernenden als deutlich lehrreicher und motivierender wahrgenommen wurde und auch bei der objektiven Evaluation des Wissenszuwachses positive Ergebnisse liefert. Damit haben wir ein lernerzentriertes Lehrformat evaluiert, dass die Studierenden praxisnah auf Herausforderungen des zukünftigen Arbeitslebens vorbereitet und gleichzeitig das Potenzial hat, motivierte Nachwuchskräfte für chirurgische Fachgebiete zu begeistern. Probleme zeigten sich bei der Integration eines Onlinekonzepts, da der E‑Learning-CBL-Kurs insgesamt zwar schlechter als das Präsenzformat, aber dennoch zufriedenstellend evaluiert wurde. Im Hinblick auf aktuelle Entwicklungen in der universitären Lehre aufgrund der COVID-19-Pandemie, die einen kontaktarmen studentischen Unterricht notwendig machen, eröffnen gerade onlinebasierte „Case-based-learning“-Programme neue Möglichkeiten. So war zwar die Digitalisierung frontal gehaltener Unterrichtsformate wie Vorlesungen weitgehend problemlos und ohne große Qualitätsminderung möglich, die adäquate digitale Nachbildung von Unterricht am Krankenbett stellte die Universitäten jedoch vor große Herausforderungen [18]. Hier bieten „Case-based-learning“-Formate die Möglichkeit, die medizinische Lehre auch online praxisnäher und interaktiver zu gestalten und damit auch in Zeiten der Kontaktbeschränkungen möglichst viele Studierende für chirurgische Fächer zu begeistern [19]. Die Erkenntnisse unserer Untersuchungen sollten genutzt werden, um fallbasierte Lehrformate weiter zu verbessern und gerade onlinebasierte Lehrformate noch besser an die Bedürfnisse der Lernenden anzupassen.
